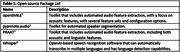# Global Research Integration Platform (GRIP): Open‐source Digital Voice Processing Toolkit

**DOI:** 10.1002/alz.091274

**Published:** 2025-01-09

**Authors:** Cody Karjadi, Huitong Ding, Edward Searls, Julia Peterson, Katherine A. Gifford, Abhishek Pratap, Ting Fang Alvin Ang, Rhoda Au

**Affiliations:** ^1^ Boston University Chobanian & Avedisian School of Medicine, Boston, MA USA; ^2^ Framingham Heart Study, Boston University Chobanian & Avedisian School of Medicine, Boston, MA USA; ^3^ Department of Neurology, Vanderbilt University Medical Center, Nashville, TN USA; ^4^ Vanderbilt Memory & Alzheimer’s Center, Vanderbilt University Medical Center, Nashville, TN USA; ^5^ University of Washington, Seattle, WA USA; ^6^ Institute of Psychiatry, King's College London, London UK; ^7^ University of Toronto, Toronto, ON Canada; ^8^ Boston University School of Public Health, Boston, MA USA; ^9^ Department of Anatomy & Neurobiology, Boston University Chobanian & Avedisian School of Medicine, Boston, MA USA; ^10^ Framingham Heart Study, Framingham, MA USA; ^11^ Department of Pharmacology, Physiology & Biophysics, Boston University Chobanian & Avedisian School of Medicine, Boston, MA USA; ^12^ Slone Epidemiology Center, Boston University Chobanian & Avedisian School of Medicine, Boston, MA USA; ^13^ Boston University School of Medicine, Boston, MA USA; ^14^ The Framingham Heart Study, Boston University School of Medicine; Boston University School of Public Health, Boston, MA USA; ^15^ Framingham Heart Study, Boston University School of Medicine, Boston, MA USA; ^16^ Boston University Alzheimer’s Disease Center, Boston University, Boston, MA USA; ^17^ Alzheimer’s Disease Research Center, Boston University Chobanian & Avedisian School of Medicine, Boston, MA USA; ^18^ Department of Epidemiology, Boston University School of Public Health, Boston, MA USA; ^19^ Department of Neurology, Boston University Chobanian & Avedisian School of Medicine, Boston, MA USA; ^20^ Boston University Chobanian & Avedisian School of Medicine and School of Public Health, Boston, MA USA; ^21^ The Framingham Heart Study, Framingham, MA USA; ^22^ Department of Anatomy and Neurobiology, Neurology and Medicine, Framingham Heart Study, BU Alzheimer's Disease Research Center, Boston University Chobanian & Avedisian School of Medicine, Boston, MA USA

## Abstract

**Background:**

Producing speech is a cognitively complex task and can be collected through devices such as handheld recorders, tablets, and smartphones. Digital voice data can also capture information at a granular millisecond‐level precision and serve as a widespread tool to collect cognitively relevant data in almost any diverse real‐world environments.

Digital voice recordings of spoken responses to neuropsychological test questions have been collected through the Framingham Heart Study (FHS) since 2005. The methods to analyze voice recordings were initially labor and time‐intensive approaches that were significant barriers to fully realizing the scientific objective of using speech and language as an alternative approach to cognitive assessment.

**Methods:**

Through a collaboration with the Global Research Integration Platform (GRIP) and FHS, we leveraged existing open–source tools to create a digital voice processing toolkit that can be used by the general scientific community. GRIP is modularizing this toolkit to allow for seamless integration into various sites worldwide with low‐to‐high levels of technical experience.

Table 1 lists the initial open‐source tools we tested on 9,253 audio recordings collected on 5,399 FHS participants. Each open‐source tool has a Python Github repository that we leveraged.

**Results:**

With minimal manual intervention, we generated prosodic, spectral, cepstral, and sound quality features from the ComParE‐2016 feature set via openSMILE. We produced 65 LLDs (low‐level descriptors) every 10 milliseconds over a 60‐millisecond window and 6373 features generated by applying several statistical functionals to the LLDs.

We segmented speakers via pyannote.audio and analyzed 9 acoustic and linguistic PRAAT features and 6373 openSMILE features in the context of a cognitive status classification task. Via Whisper, we generated timestamped transcriptions from both FHS and additional U.S. and international cohorts. We have noted difficulties in language detection and decreased transcription performance for non‐English speakers and English speakers with accents.

We have used these data in numerous studies relating digital voice to AD related outcomes.

**Conclusion:**

Digital voice is a prime candidate for scalable collection of cognitively relevant information. The modularized toolkit being developed will provide scaled non‐proprietary post‐processing of digital voice data that can be seamlessly integrated by users worldwide with low‐to‐high levels of technical experience.